# Supplementary material for: Comparison of Different Invasive and Non-Invasive Methods to Characterize Intestinal Microbiota throughout a Production Cycle of Broiler Chickens
Source: Microorganisms. 2019 Oct 10;7(10):431. doi: 10.3390/microorganisms7100431 (PMC6843853; doi:10.3390/microorganisms7100431)
Supplement: Supplementary file 1 [file microorganisms-07-00431-s001.pdf]

Supplemental Materials

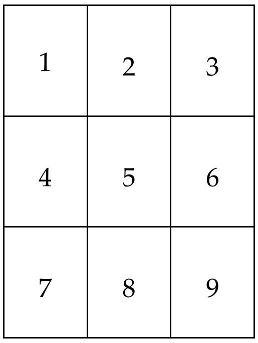

Figure S1. Map of the poultry houses: both poultry houses were divided into nine fictive sections. Boot socks samples and cecal droppings were collected in the corners (sections 1, 3, 7 and 9), and the middle of the poultry house (section 5).

a.

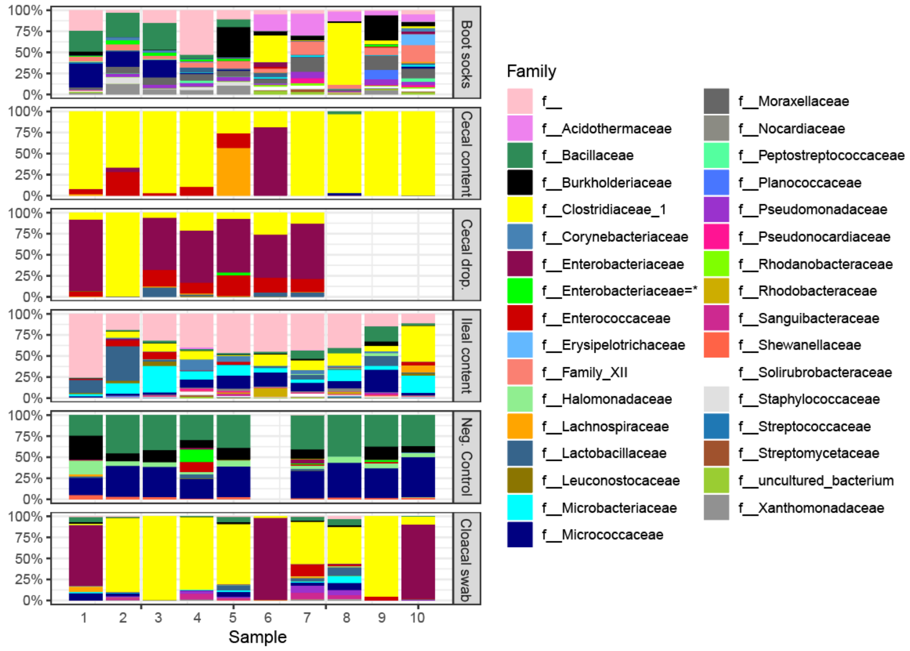

b.

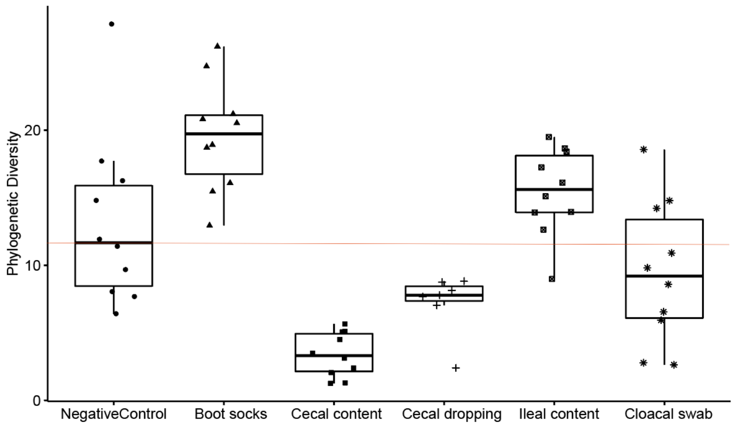

Figure S2. Results of all samples collected on day 0 and the negative control samples. a. The barplot shows the relative abundance of *Bacillaceae, Burkholderiaceae, Halomonadaceae, Micrococcaceae,* and *Shewanellaceac* in the nine negative control samples. Although overall compositional profiles were

largely different from those of negative control samples (Figure S2), those families were also abundantly present (>10%) in the boot sock and ileal samples, and therefore all samples of this day were excluded for further analysis. In the cloacal swabs, the families *Bacillaceae* and *Micrococcaceae* were present, but only at relatively low abundances (3%, Table S2), and these were therefore not excluded from the analyzed dataset. After day 0, those five families were still detected in some samples, but at low (>1%) abundance only (Table S2). b. This plot shows that phylogenetic diversity in samples from day 0 was higher in boot socks and ileal content than in the negative controls. This indicates that those samples are not suitable for analyses and were therefore excluded from the dataset.

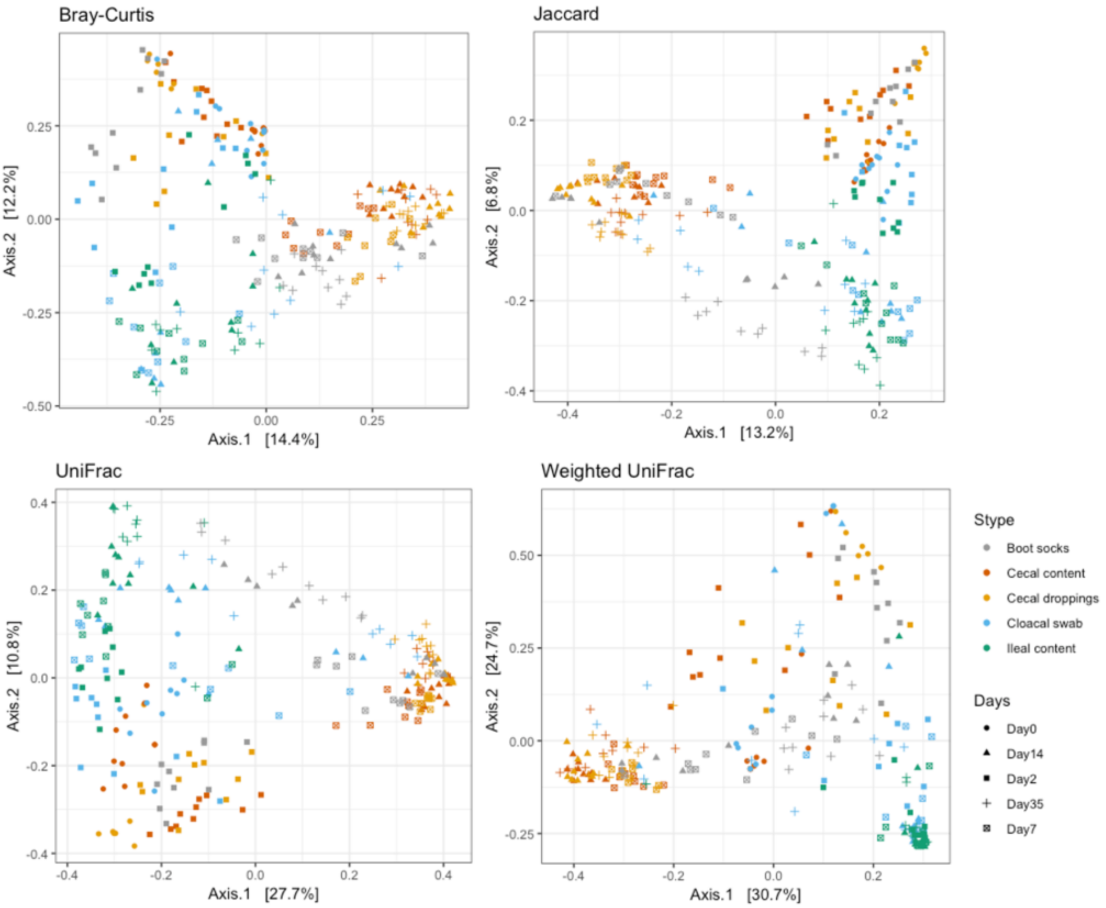

Figure S3. Principal coordinate plots (PCoA) based on Bray-Curtis, Jaccard, unweighted UniFrac and weighted UniFrac distances between cecal and ileal content, cloacal swabs, cecal droppings and boot socks. Different colors indicate different sample types, different shapes indicate different days.

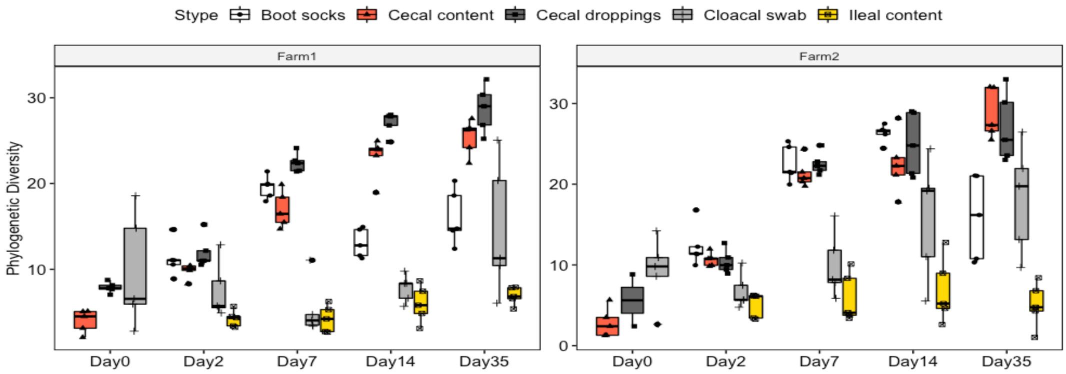

Figure S4. Phylogenetic diversity across farms within sample type.

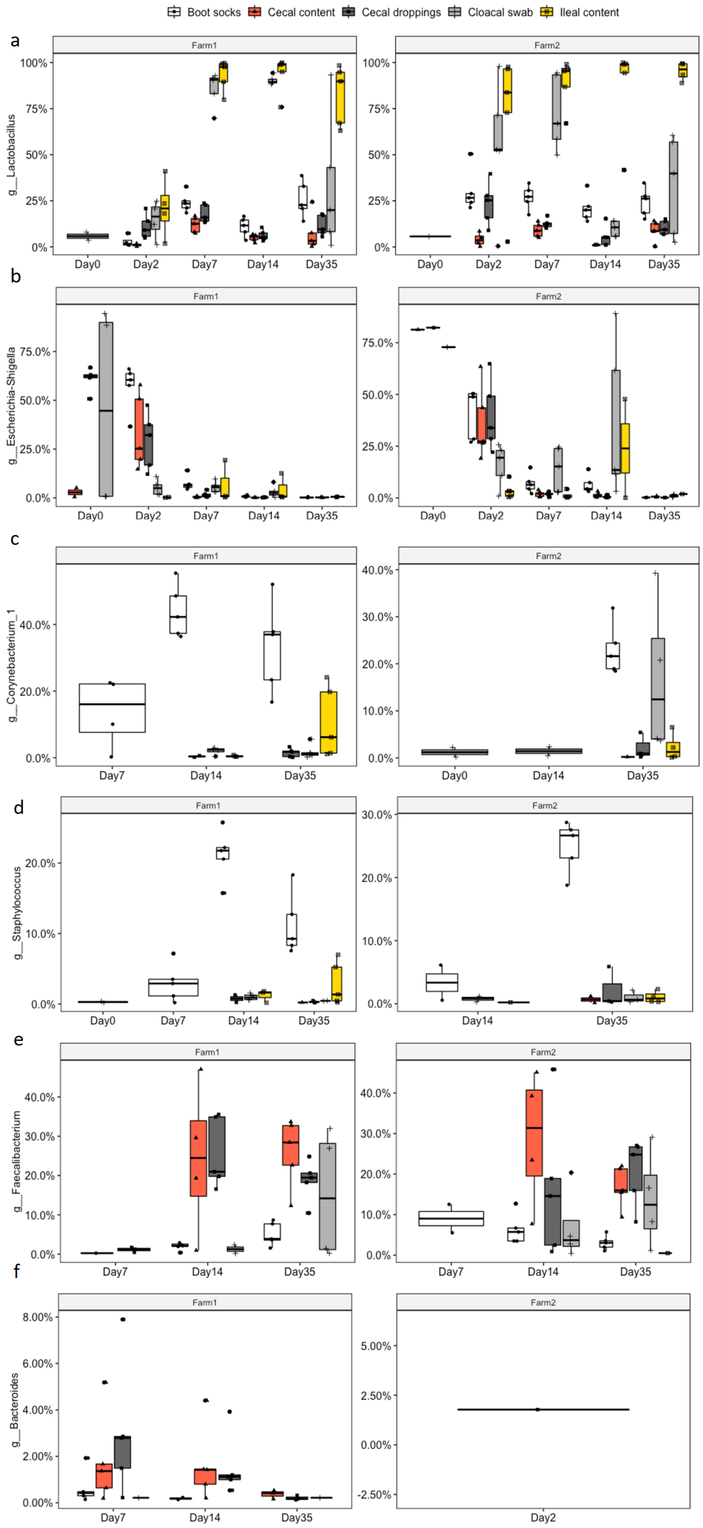

Figure S5. A selection of genera that were shown to be significantly different in relative abundance across sample types and ages (Based on Table S3, Wilcoxon rank-sum test, adjusted p-values are corrected p-values for multiple testing, BH, p-value <0.05). a. relative abundance *Lactobacillus*, b. relative abundance *Escherichia-Shigella* c. relative abundance *Corynebacterium*, d. relative abundance *Staphylococcus*, e. relative abundance *Faecalibacterium*, f. relative abundance *Bacteroides*

Table S1. Alpha diversity across sample types, age and farms based on pairwise comparisons using Wilcoxon rank sum test corrected for multiple testing with Benjamini-Hochberg.

**All ages**	Boot socks	Cecal content	Cecal droppings	Ileal content
Cecal content	0.699	-	-	-
Cecal droppings	0.136	0.080	-	-
Ileal content	< 2e-16	2.4e-07	9.3e-15	-
Cloacal swabs	3.8e-06	0.002	1.2e-06	0.001

**Age 0**	Boot socks	Cecal content	Cecal droppings	Ileal content
Cecal content	n.a.	-	-	-
Cecal droppings	n.a.	0.007	-	-
Ileal content	n.a.	n.a.	n.a.	-
Cloacal swabs	n.a.	0.007	0.417	n.a.

**Age 2**	Boot socks	Cecal content	Cecal droppings	Ileal content
Cecal content	0.051	-	-	-
Cecal droppings	0.436	0.159	-	-
Ileal content	3.6e-05	3.6e-05	3.6e-05	-
Cloacal swabs	0.004	0.015	0.006	0.054

**Age 7**	Boot socks	Cecal content	Cecal droppings	Ileal content
Cecal content	0.184	-	-	-
Cecal droppings	0.079	0.004	-	-
Ileal content	2.2e-05	2.2e-05	2.2e-05	-
Cloacal swabs	2.2e-05	7.2e-05	2.2e-05	0.190

**Age 14**	Boot socks	Cecal content	Cecal droppings	Ileal content
Cecal content	0.684	-	-	-
Cecal droppings	0.026	0.048	-	-
Ileal content	1.1e-04	5.4e-05	5.4e-05	-
Cloacal swabs	0.011	0.005	1.1e-04	0.044

**Age 35**	Boot socks	Cecal content	Cecal droppings	Ileal content
Cecal content	2.2e-05	-	-	-
Cecal droppings	2.2e-05	0.760	-	-
Ileal content	2.2e-05	2.2e-05	2.2e-05	-
Cloacal swabs	0.971	4.1e-04	4.1e-04	4.1e-04

**Farm 1 – all ages**	Boot socks	Cecal content	Cecal droppings	Ileal content
Cecal content	0.692	-	-	-
Cecal droppings	0.109	0.097	-	-
Ileal content	1.5e-10	4.3e-05	6.2e-10	-

Cloacal swabs	0.001	0.012	2.0e-05	0.015

**Farm 2 – all ages**	Boot socks	Cecal content	Cecal droppings	Ileal content
Cecal content	0.560		-	
Cecal droppings	0.774	0.392	-	-
Ileal content	6.5e-09	3.9e-04	6.8e-07	-
Cloacal swabs	6.0e-04	0.058	0.002	4.0e-04

Table S2. The relative abundance on family level across sample type and age.

**Day0**	**Cecal content**	**Cecal droppings**	**Cloacal swab**
f__		<0.000%	0.502%
f__Bacillaceae	0.152%		2.593%
f__Bifidobacteriaceae		<0.000%	0.082%
f__Burkholderiaceae		<0.000%	1.057%
f__Clostridiaceae_1	75.804%	22.631%	51.590%
f__Corynebacteriaceae		<0.000%	0.488%
f__Defluviitaleaceae			0.076%
f__Devosiaceae		<0.000%	0.251%
f__Dietziaceae		<0.000%	0.025%
f__Enterobacteriaceae	4.253%	59.989%	29.005%
f__Enterococcaceae	9.231%	14.201%	1.076%
f__Erysipelotrichaceae		0.025%	<0.000%
f__Halomonadaceae	0.022%	<0.000%	0.388%
f__Lachnospiraceae	10.347%	0.464%	1.037%
f__Lactobacillaceae	0.076%	2.283%	1.817%
f__Leuconostocaceae		0.304%	0.057%
f__Methanosaetaceae		<0.000%	0.084%
f__Microbacteriaceae		<0.000%	1.559%
f__Micrococcaceae	0.158%	0.034%	3.132%
f__Peptostreptococcaceae		0.069%	0.042%
f__Pseudomonadaceae			1.825%
f__Rhizobiaceae		<0.000%	0.151%
f__Rhodobacteraceae		<0.000%	0.052%
f__Rikenellaceae		<0.000%	0.015%
f__Sanguibacteraceae		<0.000%	2.213%
f__Shewanellaceae	0.008%	<0.000%	0.162%
f__Staphylococcaceae			0.048%
f__Streptococcaceae			0.099%
f__Xanthomonadaceae		<0.000%	0.435%

**Day 2**	**Boot socks**	**Cecal content**	**Cecal droppings**	**Ileal content**	**Cloacal swab**
f__	0.548%	0.137%	0.278%	0.066%	0.346%
f__Acidothermaceae	0.064%				

f__Actinospicaceae	0.014%				
f__Bacillaceae	0.071%			0.063%	0.028%
f__Bacteroidaceae			0.143%		
f__Burkholderiaceae	0.058%				0.014%
f__Carnobacteriaceae					0.020%
f__Caulobacteraceae	0.035%				
f__Clostridiaceae_1	0.620%	5.192%	3.706%	0.411%	0.736%
f__Enterobacteriaceae	49.059%	33.840%	37.221%	2.057%	12.474%
f__Enterococcaceae	11.274%	6.281%	12.275%	10.541%	6.937%
f__Erysipelotrichaceae	0.861%	1.367%	2.400%		0.100%
f__Family_XII	0.380%				
f__Lachnospiraceae	4.973%	33.485%	14.538%	0.027%	7.173%
f__Lactobacillaceae	22.456%	8.891%	19.245%	71.607%	55.976%
f__Leuconostocaceae	0.675%	0.615%	0.407%	0.115%	0.107%
f__Microbacteriaceae				0.060%	0.015%
f__Micrococcaceae	0.103%			0.102%	0.042%
f__Moraxellaceae	0.092%				
f__P5D1-392	0.080%	0.003%	0.053%		0.083%
f__Paenibacillaceae		0.171%	0.273%		
f__Peptostreptococcaceae		1.059%	0.947%		0.036%
f__Planococcaceae	0.175%		0.189%		0.142%
f__Pseudomonadaceae	0.018%				0.027%
f__Pseudonocardiaceae	0.019%				
f__Rhizobiaceae	0.049%				
f__Ruminococcaceae		1.689%	0.289%		0.120%
f__Sanguibacteraceae					0.045%
f__Sphingobacteriaceae	0.016%				
f__Streptococcaceae	8.344%	7.271%	8.037%	14.951%	15.581%
f__Streptomycetaceae	0.018%				
**Day 7**	**Boot socks**	**Cecal content**	**Cecal droppings**	**Ileal content**	**Cloacal swab**
f__	1.432%	2.245%	2.296%	0.021%	0.084%
f__Aerococcaceae	2.119%				
f__Anaeroplasmataceae			0.066%		
f__Bacillaceae	0.050%	0.097%	0.007%		0.085%
f__Bacteroidaceae	0.342%	1.495%	2.377%		0.016%
f__Bifidobacteriaceae	0.494%	0.295%	0.461%	0.035%	
f__Brevibacteriaceae	0.071%				
f__Burkholderiaceae		0.006%	0.018%		0.024%
f__Clostridiaceae_1	0.031%				0.315%
f__Clostridiales_vadinBB60_group		0.382%	0.752%		
f__Corynebacteriaceae	5.577%				
f__Dermabacteraceae	0.180%				
f__Eggerthellaceae	0.010%	0.252%	0.343%		
f__Enterobacteriaceae	8.294%	0.789%	1.939%	2.572%	10.218%
f__Enterococcaceae	4.122%	0.535%	0.501%	3.209%	4.557%

f__Erysipelotrichaceae	2.432%	1.561%	3.297%	0.093%	0.245%
f__Family_XII	0.089%				
f__Family_XIII		0.023%			
f__Lachnospiraceae	34.870%	67.523%	59.252%	2.065%	4.053%
f__Lactobacillaceae	25.922%	11.223%	16.437%	91.437%	78.107%
f__Leuconostocaceae	4.094%				
f__Microbacteriaceae				0.022%	
f__Micrococcaceae		0.018%	0.020%		0.108%
f__Moraxellaceae	0.132%				
f__Peptostreptococcaceae	<0.000%	0.170%	0.028%	<0.000%	0.062%
f__Planococcaceae	0.094%			0.116%	0.131%
f__Rikenellaceae	0.041%				
f__Ruminococcaceae	7.214%	12.764%	12.018%	<0.000%	0.581%
f__Staphylococcaceae	1.486%				
f__Streptococcaceae	0.800%			0.431%	1.413%
f__uncultured_bacterium	0.103%	0.621%	0.187%		
**Day 14**	**Boot socks**	**Cecal content**	**Cecal droppings**	**Ileal content**	**Cloacal swab**
f__	0.892%	1.772%	1.923%	0.175%	0.676%
f__Aerococcaceae	4.467%		0.010%		
f__Anaeroplasmataceae		0.035%	0.079%		
f__Bacillaceae	0.963%	0.680%	0.182%	0.075%	0.421%
f__Bacteroidaceae	0.049%	0.580%	1.326%		
f__Bifidobacteriaceae	0.526%	0.900%	2.330%	0.052%	0.534%
f__Brevibacteriaceae	0.100%				
f__Burkholderiaceae				0.022%	0.130%
f__Christensenellaceae		0.019%	0.066%		
f__Clostridiaceae_1	0.106%			0.077%	2.482%
f__Clostridiales_vadinBB60_group		2.037%	0.887%		
f__Corynebacteriaceae	26.691%		0.072%	0.187%	6.024%
f__Defluviitaleaceae			0.020%		
f__Dermabacteraceae	1.477%	<0.000%	<0.000%	<0.000%	<0.000%
f__Eggerthellaceae	0.020%	0.457%	0.610%	0.005%	0.077%
f__Enterobacteriaceae	3.177%	0.590%	0.307%	3.865%	24.475%
f__Enterococcaceae	1.241%	0.023%	0.179%	0.388%	8.246%
f__Erysipelotrichaceae	0.878%	2.731%	2.699%		0.845%
f__Family_XII	0.140%				
f__Family_XIII		0.026%			
f__Halomonadaceae					0.013%
f__Lachnospiraceae	16.691%	46.080%	44.670%	0.225%	5.198%
f__Lactobacillaceae	15.738%	2.644%	5.922%	92.849%	36.865%
f__Leuconostocaceae	1.257%			0.351%	0.048%
f__Microbacteriaceae				0.223%	
f__Micrococcaceae	0.016%		0.026%	0.093%	0.405%
f__Moraxellaceae	0.027%				
f__Nocardiaceae				0.045%	

f__Pasteurellaceae					0.190%
f__Peptostreptococcaceae				0.734%	
f__Planococcaceae				0.093%	0.079%
f__Pseudomonadaceae	0.008%				
f__Rikenellaceae	0.105%	0.373%	0.305%		0.064%
f__Ruminococcaceae	9.478%	40.123%	36.432%	0.023%	9.910%
f__Sanguibacteraceae				0.024%	
f__Staphylococcaceae	15.577%		0.131%	0.495%	0.582%
f__Streptococcaceae	0.015%				2.738%
f__uncultured_bacterium	<0.000%	0.930%	1.826%	<0.000%	<0.000%
**Day 35**	**Boot socks**	**Cecal content**	**Cecal droppings**	**Ileal content**	**Cloacal swab**
f__	0.275%	1.192%	1.938%	0.021%	0.838%
f__Actinomycetaceae					13.517%
f__Aerococcaceae	2.064%		0.016%	0.192%	0.071%
f__Akkermansiaceae		0.491%	0.130%		0.023%
f__Anaeroplasmataceae		0.028%			
f__Bacillaceae	0.166%	0.096%	0.205%	0.024%	0.012%
f__Bacteroidaceae		0.131%	0.056%		0.022%
f__Bifidobacteriaceae	0.585%	6.607%	7.706%	0.143%	4.510%
f__Brevibacteriaceae	1.979%		0.099%	0.021%	0.378%
f__Burkholderiaceae		0.554%	0.482%		0.114%
f__Carnobacteriaceae	0.082%				
f__Christensenellaceae	0.063%	0.537%	0.219%		0.045%
f__Clostridiaceae_1					0.016%
f__Clostridiales_vadinBB60_group		1.575%	2.610%		0.345%
f__Corynebacteriaceae	30.414%	0.018%	1.205%	5.448%	11.652%
f__Defluviitaleaceae		0.058%	0.022%		0.014%
f__Dermabacteraceae	2.891%	0.011%	0.102%	0.224%	0.781%
f__Desulfovibrionaceae		0.032%	0.009%		
f__Dietziaceae				0.013%	
f__Eggerthellaceae		0.363%	0.796%		0.099%
f__Enterobacteriaceae	0.138%	0.223%	0.262%	0.244%	0.632%
f__Enterococcaceae	0.438%	0.028%	0.069%	0.094%	1.884%
f__Erysipelotrichaceae	0.068%	1.098%	1.170%	<0.000%	0.274%
f__Eubacteriaceae			0.008%		
f__Family_XI					0.707%
f__Family_XIII		0.007%			
f__Lachnospiraceae	6.269%	35.144%	33.227%	0.070%	11.280%
f__Lactobacillaceae	24.611%	9.451%	10.309%	90.501%	30.972%
f__Leuconostocaceae	1.254%	0.017%	0.042%	0.057%	0.089%
f__Marinifilaceae			0.008%		
f__Microbacteriaceae				0.067%	
f__Micrococcaceae	0.016%	0.048%	0.063%	0.046%	0.020%
f__Nocardiopsaceae				0.018%	0.024%
f__P5D1-392					0.012%

f__Peptococcaceae		0.012%			
f__Peptostreptococcaceae	0.342%	0.822%	0.490%	0.414%	2.830%
f__Rikenellaceae	0.113%	1.984%	1.921%		0.205%
f__Ruminococcaceae	5.824%	34.666%	33.648%	0.060%	15.887%
f__Staphylococcaceae	21.394%	0.137%	0.511%	2.085%	0.466%
f__Streptococcaceae	0.877%	0.523%	0.603%	0.260%	1.764%
f__Tannerellaceae	0.046%	1.415%	0.197%		0.028%
f__uncultured_bacterium	0.090%	2.596%	1.859%	<0.000%	0.489%
f__uncultured_Firmicutes_bacterium		0.139%	0.017%		

Table 3. Genera that were significantly different in relative abundance. The genera that were significantly different between the sample types. The results are based on differences of relative abundance tested with Wilcoxon rank-sum test. Adjusted P-values (q-val) are corrected for multiple testing with Benjamini-Hochberg, a q-value <0.05 was considered statistically significant. Difference in relative abundance is in percent points (fold change % points).

Days	Cecal content vs cecal dropping	q-val	fc %	Cecal content vs cloacal swab	q-val	fc %	Cecal content vs Boot socks	q-val	fc %	Ileal content vs Cloacal swab	q-val	fc %
2,7,14, 35	g__Lactobacillus	0.006	-7%	g__Lactobacillus	1.31e-06	-43%	g__Lactobacillus	0.005	-15%	g__Escherichia-Shigella	0.003	-7%
				g__Streptococcus	0.005	-5%	g__Corynebacterium_1	0.035	-14%	g__Faecalibacterium	0.005	-4%
				g__Enterococcus	6.42e-04	-3%	g__Staphylococcus	4.27e-01	-8%	f__Actinomycetaceae	0.035	-3%
				f__Actinomycetaceae	1.80e-04	-3%	g__Enterococcus	3.39e-04	-2%	g__Enterococcus	0.003	-2%
				g__Corynebacterium_1	4.60e-04	-2%	g__Weissella	5.38e-04	-2%	g__Corynebacterium	0.035	-2%
				g__Corynebacterium	0.018	-2%	g__Aerococcus	1.28e-04	-2%	g__[Ruminococcus]_torques_group	0.010	-2%
				g__Ruminiclostridium_5	2.51e-06	1%	g__Jeotgalicoccus	0.001	-1%	f__Lachnospiraceae	0.014	-1%
				f__Lachnospiraceae	2.51e-06	1%	g__Brachybacterium	0.010	-1%	f__Lachnospiraceae	0.009	2%
				o__Mollicutes_RF39	1.51e-04	1%	g__Butyricicoccus	0.011	1%	g__Lactobacillus	0.005	27%
				g__Butyricicoccus	1.18e-07	2%	f__Ruminococcaceae	6.12e-04	2%			
				f__Ruminococcaceae	0.010	2%	g__Blautia	1.26e-04	3%			
				g__Erysipelatoclostridium	3.23e-07	2%	f__Lachnospiraceae	0.005	3%			

				g__Subdoligranulum<br>g__Fusicatenibacter<br>g__Eisenbergiella<br>f__Lachnospiraceae<br>g__Blautia<br>f__Lachnospiraceae<br>g__[Ruminococcus]_torques_group	0.011<br>0.005<br>6.42e-06<br>0.009<br>9.78e-07<br>8.78e-09<br>8.78e-09	2%<br>3%<br>3%<br>4%<br>4%<br>9%<br>12%	f__Lachnospiraceae<br>g__[Ruminococcus]_torques_group	0.014<br>0.010	6%<br>10%			
0	No differences			No differences			n.a.			n.a.		
2	g__Lactobacillus.	0.018	-15%	g__Escherichia-Shigella	0.048	24%	No differences			No differences		
7	No differences			g__Lactobacillus<br>g__Escherichia-Shigella<br>g__Enterococcus<br>g__Streptococcus<br>g__Erysipelatoclostridium<br>g__Sellimonas<br>o__Clostridiales;f__<br>f__Lachnospiraceae	0.001<br>0.001<br>0.005<br>0.015<br>0.006<br>0.001<br>0.012<br>0.033	-69%<br>-9%<br>-5%<br>-1%<br>1%<br>2%<br>2%<br>2%	g__Lactobacillus<br>g__Escherichia-Shigella<br>g__Enterococcus	0.001<br>0.003<br>0.005	-15%<br>-6%<br>-4%	No differences		

				g__Ruminiclostridium_5	0.005	2%						
				g__Lachnoclostridium	0.001	2%						
				g__Butyricicoccus	0.001	2%						
				f__Ruminococcaceae	0.002	3%						
				g__Blautia	0.001	5%						
				f__Lachnospiraceae	0.001	8%						
				g__Eisenbergiella	0.001	8%						
				f__Lachnospiraceae	0.001	13%						
				g__[Ruminococcus]_torques_gro	0.001	17%						
14	No differences			g__Lactobacillus	0.002	-47%	g__Lactobacillus	0.003	-13%	No differences		
				g__Escherichia-Shigella	0.010	-19%	g__Staphylococcus	0.033	-11%			
				g__Enterococcus	0.005	-6%	g__Enterococcus	0.003	-1%			
				g__Streptococcus	0.038	-3%	f__Clostridiales_vadinBB60_group	0.003	2%			
				g__Candidatus_Arthromitus	0.005	-2%						
				g__Corynebacterium_1	0.018	-1%						
				o__Clostridiales;f__	0.013	1%						
				g__Sellimonas	0.002	1%						

				f__Ruminococcaceae;g__	0.005	1%						
				o__Mollicutes_RF39	0.018	1%						
				f__Lachnospiraceae	0.005	1%						
				g__[Eubacterium]_hallii_group	0.012	1%						
				g__Ruminiclostridium_5	0.002	1%						
				g__Erysipelatoclostridium	0.027	1%						
				g__Butyricicoccus	0.005	2%						
				f__Clostridiales_vadinBB60_group	0.002	2%						
				f__Ruminococcaceae;g__uncultured	0.010	2%						
				g__Fusicatenibacter	0.010	4%						
				g__Blautia	0.010	5%						
				f__Lachnospiraceae;g__	0.002	5%						
				f__Lachnospiraceae	0.002	10%						
				g__[Ruminococcus]_torques_group	0.003	15%						
35	No differences			f__Actinomycetaceae;g__	0.035	-11%	g__Corynebacterium_1	0.001	-28%	g__Faecalibacterium	0.044	-11%
				g__Corynebacterium_1	0.013	-8%	g__Staphylococcus	0.001	-18%	f__Actinomycetaceae	0.044	-11%

				g__Enterococcus	0.013	-2%	g__Lactobacillus	0.002	-17%	g__Blautia	0.044	-2%
				g__Butyricicoccus	0.041	1%	g__Jeotgalicoccus	0.001	-4%	g__Enterococcus	0.047	-2%
				g__Alistipes	0.013	2%	g__Brachybacterium	0.001	-3%	g__Subdoligranulum	0.044	-1%
				g__[Ruminococcus]_torques_group	0.013	6%	g__Brevibacterium	0.001	-2%			
							g__Aerococcus	0.001	-2%			
							g__Weissella	0.006	-1%			
							g__Ruminiclostridium_5	0.002	1%			
							g__Butyricicoccus	0.001	1%			
							g__[Eubacterium]_hallii_group	0.008	1%			
							f__Ruminococcaceae	0.002	1%			
							f__Clostridiales_vadinBB60_group	0.002	2%			
							g__Alistipes	0.002	2%			
							o__Mollicutes_RF39	0.012	3%			
							f__Lachnospiraceae	0.002	3%			
							g__Blautia	0.026	5%			
							f__Lachnospiraceae	0.003	6%			
							g__[Ruminococcus]_torques_group	0.002	7%			
							g__Faecalibacterium	0.001	17%			

microorganisms
